# Perceived Parental Functioning, Self-Esteem, and Psychological Distress in Adults Whose Parents are Separated/Divorced

**DOI:** 10.3389/fpsyg.2015.01760

**Published:** 2015-11-17

**Authors:** Maria C. Verrocchio, Daniela Marchetti, Mario Fulcheri

**Affiliations:** ^1^Department of Psychological Sciences, Health and Territory, ‘G. d’Annunzio’ University of Chieti-PescaraChieti, Italy; ^2^Department of Medicine and Aging Sciences, ‘G. d’Annunzio’ University of Chieti-PescaraChieti, Italy

**Keywords:** parental separation/divorce, loyalty conflicts, parental bonding, self-esteem, psychological distress

## Abstract

**Objective:** The objective of this research was to identify retrospectively the alienating behaviors and the parental bonding that occurred in an Italian sample of adults whose had parents separated or divorced and their associations with self-esteem and psychological distress.

**Methods:** Four hundred seventy adults in Chieti, Italy, completed an anonymous and confidential survey regarding their childhood exposure to parental alienating behaviors (using the Baker Strategy Questionnaire), quality of the parent–child relationship (using Parental Bonding Instruments), self-esteem (using Rosenberg Self-Esteem Scale), and global psychological distress (using Global Severity Index of Symptom Checklist-90-Revised).

**Results:** About 80% of the sample reported some exposure to parental alienating behaviors; about 65–70% of the sample has perceived non-optimal parenting by mother and by father; individuals who experienced affectionless control (low care and high overprotection) reported significantly higher exposure to parental loyalty conflict behaviors. Overall rates of reported exposure to low care, and overprotection and parental loyalty conflict behaviors were statistically significantly associated with self-esteem as well as the measure of current psychological distress. Results revealed that exposure to parental loyalty conflict behaviors and self-esteem were associated with psychological distress over and above the effects of parental bonding and age.

**Conclusion:** The pattern of findings supports the theory that children exposed to dysfunctional parenting, and with low self-esteem are at risk for their long-term psychological functioning. Implications for health policy changes and strengthening social services are discussed.

## Introduction

Over the last 15 years, Italy has experienced a great increase of separations and divorces. From 1995 to 2011, separations increased by 68.8% and the rate of divorces nearly doubled. Official statistics show that in 2012 the separations were 88,288 and 51,319 divorces and that the children involved were 112.253 in separations and 53.553 in divorces. Slightly less than half (48.7%) of separations and one–third (33.1%) of divorces involve marriages with at least one child less than 18 years (ISTAT, 2014). The prevalence and consequences of marital instability and divorce in Italy have stimulated a large interest from a host of public and private sectors, including policy makers, scholars and practitioners, women’s and fatherhood associations.

In the Italian courts always more frequently separations and divorces are characterized by high and persistent conflicts. Individual problems and psychological distress during marital dissolution may interfere with parenting practices and affect the parent–child relationship. Mothers in high conflict marriages are reported to be less warm, more rejecting, and use harsher discipline, and fathers withdraw more and engage in more intrusive interactions with their children compared with parents in low-conflict marriages (Heatherington and Stanley-Hagan, 1999; Krishnakumar and Buehler, 2000).

Divorce has several direct and indirect economic consequences that are absorbed not only by individuals, but also by communities and governments. Health professionals and social workers offer notable support to families who are in high conflict to achieve agreement between parents and to decrease the effect of disruption on the quality of parent–child relationships during marital dissolution.

Moreover, following separation or divorce, in some instances of parental conflict, the child may be asked or expected to take action (as opposed to being a passive recipient of one parent’s negative view of the other parent) and may respond by aligning with one parent, sometimes referred to as triangulation in the family systems literature (Minuchin, 1974). Research on family postdivorce shows that high and persistent conflict is more likely to be destructive when parents use their children to express their anger and are verbally and physically aggressive (Kelly, 2000; Johnston et al., 2005). Parents put their children in the middle by asking children to carry hostile messages, by denigrating the other parent in front of the child, or by prohibiting mention of the other parent in their presence. Parental alienation (PA) is the term used to describe a range of parental behaviors that are likely to foster a child’s unreasonable and unwarranted rejection of the other parent, referred to as the “targeted parent.” From the studies, 17 specific behaviors have become known as the primary PA behaviors (Baker, 2007; Baker and Fine, 2013), such as “limiting contact between the child and the targeted parent,” “withholding love and affection from the child when the child is affectionate or positive toward the targeted parent,” “forcing the child to reject the targeted parent, telling the child that the targeted parent does not love him/her.” PA behaviors produce conflict between the child and targeted parent and create a psychological cohesion between the child and the parent engaging in these behaviors. In some cases, the child becomes so affected by these PA behaviors that s/he will cut off all contact with the targeted parent. In this context of court-mandated, reunification efforts attempted by mental health professionals and social workers often have a high failure rate. While the targeted parent is highly motivated to treatment, the alienating parent–child dyad is highly motivated to see treatment fail. This is because for them, the rejection of the targeted parent is not the problem, rather it is the solution to the problem (Ellis and Boyan, 2010).

The psychological base of PA – lack of empathy and the inability to tolerate the child’s separate needs and perception (Baker, 2007; Baker and Ben-Ami, 2011) – could be also the expression of a specific parenting style characterized by low care and high overprotection. Parents who engage in alienating behaviors can be cold, rejecting, disengaged as well as intrusive and psychologically controlling.

What are the negative outcomes of these dysfunctional parental behaviors? The literature on the impact of divorce on young adults shows that divorce, and inter-parental conflict can negatively affect children throughout their lifespan. Studies demonstrate that even when parents initiate a divorce when their offspring are over the age of 18, the impact can be substantial (Amato, 1994). Some of these relational patterns remain during young adulthood and may interfere with the individual’s ability to separate and become self-sufficient (O’Connor et al., 1999; Amato and Sobolewsky, 2001; Amato and Afifi, 2006). Children of divorce who feel caught between their parents’ conflict have been found to have a more problematic adjustment and internalizing problems than those who do not feel caught (Buehler and Welsh, 2009). According to Amato and Afifi (2006) youth who are involved in their parents’ conflict can feel caught between their parents and hence experience stressful loyalty conflicts and cognitive dissonance. Although such an alliance can resolve the child’s immediate feeling of being caught in the middle (Ellis and Boyan, 2010), it can entail its own set of problems. For example, Amato and Afifi (2006) suggest that when children become turned against one parent, they lose the support and guidance of the now rejected parent, and they may experience feelings of guilt and shame for rejecting and betraying the other parent. Several studies have shown significant associations between recall of exposure to parental loyalty conflicts behaviors and negative outcomes in adulthood as a low autonomy, low cooperativeness, low self-esteem, depressive symptoms, and psychological distress (Baker and Ben-Ami, 2011; Ben-Ami and Baker, 2012; Verrocchio and Baker, 2013; Bernet et al., 2015). Therefore is not simply the degree of parental conflict that seems to affect youth detrimentally following the divorce of their parents, but rather the degree to which they are drawn into their parents’ conflict (and hence likely to be exposed to PA behaviors).

Parental bonding is considered as a main developmental task that is critical for adequate functioning. Comfortable bonding with caregivers is assumed to provide the psychological foundation for healthy functioning in adulthood. Many studies have found associations of parental care and overprotection with domains of adult functioning (Ingram et al., 2001; Wark et al., 2003) and with development of psychiatric disorders (Mancini et al., 2000; Enns et al., 2002). Living with competent parents is a protective factor associated with positive outcome in children (Heatherington and Stanley-Hagan, 1999; Kelly and Emery, 2003; Verrocchio et al., 2013). When parents provide warmth, emotional support, adequate monitoring, authoritarian discipline, and maintain age-appropriate expectations, children and adolescents experience positive adjustment compared with children whose divorced parents are inattentive, less supportive, and use coercive discipline (Heatherington and Stanley-Hagan, 1999; Krishnakumar and Buehler, 2000; Krishnakumar et al., 2003). The long term outcomes of children are dependent on many factors, among them the quality of parenting they received before and after divorce, and the encapsulation in the conflict between parents that they experienced during the marriage and after divorce.

Many authors have emphasized the role of early relationships with parents in determining the way in which the children develop their beliefs about their worth as a separate, unique person and where they stand in relation to others (Sroufe, 1978). Self-esteem is a significant psychological construct and a central component of individuals’ daily experience. It refers to how individuals feel about themselves and reflects and affects their transactions with the environment and the people they encounter (Rosenberg, 1965; Kernis, 2003). Self-esteem was found to be positively related to mother and father care and negatively related to parental overprotection for people with Anglo and Vietnamese cultural backgrounds (Herz and Gullone, 1999). Other studies found that family environment, especially the problematic parental relationship, is a predictor of self-esteem (Levy-Shiff, 2001). Moreover, research on the relations between self-esteem and psychopathological symptoms, both in children and adolescents and in adults, show inverse relationships between these constructs (Yuang, 2000; Garaigordobil et al., 2008).

Another important issue is that the negative outcomes of divorce on children do not depend only on family processes, such as family dysfunction and parental conflict. Literature has demonstrated that children and adolescents vary as to their vulnerability to the effects of marital conflict (Heatherington and Stanley-Hagan, 1999; Kenyon and Koerner, 2008). Negative life events – in which we might include also separation/divorce with high conflict – may trigger psychological stress which can also be influenced by individual factors, including self-esteem (Plancherel et al., 1997). Therefore, an important question is to explore whether certain children are more or less vulnerable due to their own personal qualities. The study of these individual qualities may provide information on potential protective factors to help them even in at-risk contexts (Camisasca et al., 2013) and to direct intervention programs aimed to disrupt the intergenerational cycle of dysfunctional relationships. In the Italian context, international psychological literature is frequently used because there is a lack of national researches on some crucial issues that may have significant implications for what is in the best interest of children in divorced families who are in legal conflicts. To date, no researcher has attempted to examine how the associations between parental functioning – in terms of parental bonding and PA – and self-esteem could affect psychological distress in adults whose parents are separated/divorced. Since individual protective factors are a meaningful variables for psychological adjustment, we are interested to know the extent to which family variables (i.e., parental bonding and PA) and an individual variable (self-esteem) could predict psychological distress in adulthood.

The specific questions in the current study included: (1) What is the prevalence of recall of childhood exposure to parental loyalty conflict behaviors in an Italian population of adults whose parents had separated or divorced?; (2) What kind of parental bonding perceived adults whose parents had separated or divorced?; (3) Did recall of exposure to parental loyalty conflict behaviors vary by parental bonding?; (4) Were reports of parental bonding and recall of exposure to parental loyalty conflict behaviors associated with self-esteem?; (5) Was psychological distress predicted by parental functioning and individual self-esteem?

## Material and Methods

### Participants and Procedure

Between April and December of 2013, flyers were delivered to a variety of employment, recreational, and university settings in the southern region of Italy as well as distributed to friends and colleagues who were encouraged to forward it to others. The flyers stated that a researcher in the Clinical Psychology Laboratory at University of Chieti was “Seeking adults whose parents are separated/divorced.” Interested individuals were invited to contact the researcher via telephone or e-mail. Once individuals came to the Laboratory were asked for their willingness to take part in a research study related to parental relationships. Those who were interested were informed of the voluntary nature of their participation and they right to withdraw at any time. All participants received and signed an informant consent. Individuals who provided consent were escorted to a private area where they could sit and complete the questionnaire packet. The packet contained two demographic questions that would provide certain information about participants regarding specific inclusion/exclusion criteria. Specifically, only those individuals were selected for the final sample who experienced parental separation/divorce and who had both parents alive until the age of at least 12 years.

A non-random convenience sample of 497 participants was recruited. Nineteen cases were eliminated due to incomplete data, and an additional eight participants were excluded because they only had one parent. Thus, the final sample was made up of 470 adults whose parents are separated/divorced residing in southern Italy. Mean age of participants was 25.4 (*SD* = 6.13), and 55.5% were female; 76.4% of the participants had a high school degree or less, 41% had a job, and 40% were students. Mean age at first parental separation was 12.7 (*SD* = 6.10). The protocol was realized according to the ethical guidelines of the Italian Association of Psychology (AIP) and was approved by the local ethics committee.

### Measures

The paper and pencil measures consisted of a series of demographic questions (age, age at first parental separation/divorce, gender, level of education, and employment) and a series of standardized questionnaires, four of which were examined for this study.

Baker Strategy Questionnaire (BSQ; Baker and Chambers, 2011). The BSQ is a 20-item measure comprised of a list of 19 specific behaviors and one general behavior that parents might engage in to induce loyalty conflict in their child. The respondents answered separately for mother and father on a five- point scale from never (0) to always (4). Total scores could range from 0 to 80 for each parent. For the mother total scores ranged from 0 to 80 (Mean = 13.5, *SD* = 14.7) and for the father total scores ranged from 0 to 66 (Mean = 10.6, *SD* = 12.4). In the present study, the measure demonstrated high internal consistency for the mother and for father (α = 0.94). Scores across parents were also combined to create a “total exposure to PA measure” which could range from 0 to 160 and did range from 0 to 122 (Mean = 23.8, *SD* = 23.7).

Parental Bonding Instrument (PBI; Parker et al., 1979; Scinto et al., 1999). The quality of the parent–child relationship was measured with the Parental Bonding Instrument, a widely used research tool for assessing adult retrospective accounts of two dimensions of the parent–child relationship: care (12 items) and over-protectiveness (13 items). Respondents completed the scale twice, once for mother, once for father. The Care scale consists of items tapping warmth, understanding, and acceptance (e.g., “Enjoys talking things over with me,” “Makes me feel better when I’m upset”). The Overprotection scale measures control, intrusiveness, and encouragement of dependence (e.g., “Tries to control everything I do,” “Invades my privacy”). Total scale scores could range from 0 to 36 (Care scale) and from 0 to 39 (Overprotection scale). The mean scores in this sample were: mother care = 25.22, *SD* = 7.7, mother overprotection = 13.81, *SD* = 6.7, father care = 20.53, *SD* = 8.4, father overprotection = 12.10, SD = 6.6. Cronbach’s alpha for the four scales were: mother care = 0.93, mother overprotection = 0.85, father care = 0.92, father overprotection = 0.83. Additionally, scores can be assigned to four quadrants reflecting variations in care and control: (1) high care/low control = optimal parenting, (2) high care/high control = affectionate constraint, (3) high control/low care = affectionless control and (4) low care/low control = neglectful. Assignment to ‘high’ and ‘low’ categories is based on the following cut-off scores: mothers, 27 for care and 14 for protection; fathers, 24 for care and 13 for protection (Parker et al., 1979). Overprotection and Care scales were created for each parent and then summed to create an overall Overprotection and Care index.

Rosenberg Self-Esteem Scale (RSES; Rosenberg, 1965; Prezza et al., 1997). Self-esteem was assessed with the 10-item self-report Rosenberg Self-Esteem Scale in which each item was rated on a four-point Likert scale from strongly disagree (0) to strongly agree (4). Total scores were created by summing the 10 items after reverse coding. In this study the summary score ranged from 16 to 40 (Mean = 29.4, *SD* = 4.8) and had an internal consistency coefficient of 0.82.

Symptom Checklist-90-Revised (SCL-90-R; Derogatis, 1994; Prunas et al., 2012). This is a self-report questionnaire applied as a psychiatric case-finding instrument, as a measure of symptom severity, and as a descriptive measure of psychopathology in different patient populations. The SCL-90 is intended to measure symptom intensity on nine different subscales and three global indexes of distress. The 90 items of the questionnaire are scored on a five-point Likert scale from none (0) to extreme (4), indicating the rate of occurrence of the symptom during the time period in question. The instrument’s Global Severity Index (GSI) is created as the mean value of all of the items and ranges from 0 to 4. The GSI is the most often used SCL-90-R global index when a single measure of psychological distress is warranted [40]. In the current study only the GSI was used as a measure of global psychological distress. In this sample the GSI ranged from 0 to 3.26 (Mean = 0.74, *SD* = 0.58), with a Cronbach’s alpha of 0.98.

## Results

All data were checked for invalid values, out-of-range data, and counting of missing values. Missing items were replaced with the individual’s mean item score of the completed items as recommended by Downey and King (1998). In addition, the scores were checked for normality, and parametric tests were then applied.

Descriptive statistics were calculated for the sample. Preliminary analyses were conducted to test whether any of the demographic variables (age, age at first parental separation, gender, and educational level) were significantly associated with the dependent measure. Only participant’s age was significantly associated with GSI score; consequently it was included in subsequent analyses.

### What is the Prevalence of Recall of Childhood Exposure to Parental Loyalty Conflict Behaviors in an Italian Population of Adults Whose Parents Separated or Divorced?

To address the first research question about the prevalence of childhood exposure to parental loyalty conflict behaviors, the proportion of respondents who endorsed each of the 20 behaviors was calculated with frequency distributions (see **Table 1**).

**Table 1:** Frequency distribution of endorsement of 20 specific loyalty conflict behaviors.

Behavior	*N*	%
Made negative comments	378	81.4
Discomfort at other parent	288	62.1
Confided in child	277	59.7
Encouraged reliance on him/herself	255	55.5
Upset at child’s affection with other parent	244	53.4
Required favoritism of child	247	53.3
Tried to turn against other parent	216	46.1
Asked child to keep secrets	208	44.7
Made child choose	189	40.7
Limited contact	184	39.8
Fostered anger/hurt at other parent	167	35.8
Encouraged disregard of other parent	162	35.2
Asked child to spy	159	34.4
Said parent was unloving	152	32.9
Said parent was unsafe	135	29.1
Hard to be with extended family	134	29.0
Made communication difficult	124	26.6
Called other parent by first name	82	17.8
Withheld or blocked messages	71	15.3
Referred to new spouse mom/dad	46	9.9

As can be seen, all 20 behaviors were endorsed by at least some participants. Looking at the proportion of endorsers of each behavior, it can be seen that 19 of the 20 items were endorsed by at least 10% of the sample. Two items were endorsed by over 60% of the sample (made negative comments, indicated discomfort about other parent). Seven of the behaviors were endorsed by between 40 and 60% of the respondents (made child choose, asked child to keep secrets, tried to turn against other parent, required favoritism of child, upset child affectionate with other parent, encouraged reliance on himself or herself, confided in child). Eight of the PA behaviors were endorsed by 20 and 40% of the respondents (made communication difficult, hard to be with extended family, said parent was unsafe, said parent was unloving, asked child to spy, encouraged disregard of other parent, made it fostered anger/hurt with other parent, limited contact with other parent). Three behaviors were endorsed by under 20% of the respondents (called other parent by first name, withheld or blocked messages, and referred to the new spouse as Mom or Dad).

### What Kind of Parental Bonding have Perceived Adults Whose Parents had Separated or Divorced?

To address the second research question we examined the parental Care and Overprotection data. The distribution of cases across the four possible parenting types revealed that for mothers 151 (35%) of the sample were in the “optimal parenting” quadrant, 83 (19.2%) were in the “affectionate constraint” quadrant; 113 (26.2%) were in the affectionless control quadrant, and 85 (19.7%) were in the neglectful parenting quadrant. For fathers, the percentages were 29.4% for optimal parenting, 14.5% for affectionate constraint, 28.5% for affectionless control, and 26.4% were in the neglectful parenting quadrant.

### Did Recall of Exposure to Parental Loyalty Conflict Behaviors Vary by Parental Bonding?

In order to examine the relationship between exposure to parental loyalty conflict behaviors and parental bonding, we examined mean differences in BSQ scores by PBI quadrants (optimal parenting, affectionate constraint, affectionless control, neglectful parenting). One-way ANOVA’s were conducted to test the hypothesis that perceived lack of care and overprotection during childhood would be associated with exposure to parental loyalty conflict behavior by mothers and by fathers. Mean scale scores are presented in **Table 2**.

**Table 2:** Means of parental alienation behaviors by PBI bonding quadrants of parents.

	By Mother			By Father		
Parenting	*n*	*M (SD)*	95% CI	*n*	*M (SD)*	95% CI
Optimal	151	8.4 (10.5)	6.75, 10.13	127	6.9 (8.1)	5.53, 8.37
Affectionate	83	8.4 (9.7)	6.24, 10.49	63	6.4 (7.6)	4.47, 8.32
Affectionless	113	22.7 (17.8)	19.39, 26.04	123	18.2 (16.4)	15.25, 21.10
Neglectful	85	15.0 (14.7)	11.87, 18.20	114	8.9 (9.9)	7.02, 10.73

*CI, confidence interval. Optimal (parenting); affectionate (constraint); affectionless (control); neglectful (parenting).*

The results indicated significant differences across PBI quadrants (mother) for exposure to maternal loyalty conflict behaviors [*F*(3,431) = 28.975, *p* < 0.001]. *Post hoc* test Bonferroni revealed that, compared to individuals who reported optimal parenting and affectionate constraint, individuals who experienced affectionless control reported significantly higher exposure to maternal loyalty conflict behaviors. Moreover, individuals who reported neglectful parenting had significantly higher recall of exposure to maternal loyalty conflict behaviors compared to participants who reported optimal parenting and affectionate constraint, and lower recall compared to adults who experienced maternal affectionless control.

For paternal parenting, differences across PBI quadrants (father) on measures of exposure to paternal loyalty conflict behaviors were significant [*F*(3,426) = 25.702, *p* < 0.001]. *Post hoc* test Bonferroni revealed that, compared to individuals who reported optimal parenting, affectionate constraint, and neglectful parenting, individuals who experienced affectionless control reported significantly higher recall of exposure to paternal loyalty conflict behaviors.

### Were Reports of Parental Bonding and Recall of Exposure to Parental Loyalty Conflict Behaviors Associated with Self-esteem?

Pearson correlations were conducted between RSES score and both the PBI and BSQ overall indexes. These data are presented in **Table 3**. As can be seen parental care, parental overprotection, and recall of exposure to parental loyalty conflict behaviors were statistically significantly associated with self-esteem. Higher care scores, lower overprotection scores, and lower recall of exposure to parental loyalty conflict behaviors were all associated with higher self-esteem scores.

**Table 3:** Bivariate correlations for study variables.

	1	2	3	4	5	6
(1) SCL-90-R – global severity index	1	-0.114^∗^	-0.244^∗∗^	0.193^∗∗^	0.239^∗∗^	-0.537^∗∗^
(2) Age		1	-0.203^∗∗^	0.144^∗∗^	0.042	0.132^∗∗^
(3) PBI – parental care			1	-0.313^∗∗^	-0.410^∗∗^	0.273^∗∗^
(4) PBI – parental overprotection				1	0.251^∗∗^	-0.230^∗∗^
(5) BSQ – parental alienation					1	-0.232^∗∗^
(6) RSES – self-esteem						1

*SCL-90-R, Symptom Checklist-90-Revised; PBI, Parental Bonding Instrument; BSQ, Baker Strategy Questionnaire; RSES, Rosenberg Self-Esteem Scale.*

*^∗^*p* < 0.05. ^∗∗^*p* < 0.01.*

### Was Psychological Distress Predicted by Parental Functioning and Individual Self-esteem?

To address the last research question we began with Pearson correlations between age, PBI and BSQ overall indexes, RSES and GSI scores. Results revealed that GSI score was statistically positively associated with parental overprotection (*r* = 0.193, *p* < 0.01) and recall of exposure to parental loyalty conflict behaviors (*r* = 0.239, *p* < 0.01), and was statistically negatively associated with age (*r* = -0.114, *p* < 0.05), parental care (*r* = -0.244, *p* < 0.01), and self-esteem (*r* = -0.537, *p* < 0.01).

All of these variables were entered into hierarchical regression to test whether psychological distress was predicted by parental functioning (in terms of care, overprotection and exposure to parental loyalty conflict behaviors) and by self-esteem. In regression a GSI score was the dependent variable and parental care, parental overprotection, and age were entered as a first block, recall of exposure to parental loyalty conflict behaviors was added in the second block followed by self-esteem in the third block. The data are presented in **Table 4**.

**Table 4:** Hierarchical regression analysis predicting global severity index (GSI) from familiar and individual variables.

Predictor	Δ *R^2^*	β
Step 1	7.8^∗∗∗^	
PBI – parental care		-0.220^∗∗∗^
PBI – parental overprotection		0.114^∗^
Age		-0.150^∗∗^
Step 2	1.9^∗∗^	
PBI – parental care		-0.161^∗∗^
PBI – parental overprotection		0.090
Age		-0.139^∗∗^
BSQ – parental alienation		0.155^∗∗^
Step 3	19.4^∗∗∗^	
PBI – parental care		-0.056
PBI – parental overprotection		0.022
Age		-0.046
BSQ – parental alienation		0.101^∗^
RSES – self-esteem		-0.474^∗∗∗^
Total *R*^2^	29.1^∗∗∗^	
*n* = 423		

*PBI, Parental Bonding Instrument; BSQ, Baker Strategy Questionnaire; RSES, Rosenberg Self-Esteem Scale. ^∗^*p* < 0.05, ^∗∗^*p* < 0.01, ^∗∗∗^*p* < 0.001.*

The hierarchical regression revealed that at Block 1 all of the variables contributed significantly to the regression model, *F*(3,419) = 11.77, *p* < 0.001 and accounted for 7.8% of the variation in global psychological distress. Introducing parental loyalty conflict behaviors (Block 2) explained an additional 1.9% of variation in global psychological distress and this change in *R*^2^ was significant, *F*(4,418) = 11.23, *p* < 0.001. Each of the variables entered in Block 1, with the exception of parental overprotection, remained significant predictors once parental loyalty conflict behaviors were added to the model. The addition of self-esteem to the regression model (Block 3) explained an additional 19.7% of the variation in global psychological distress. The change in *R*^2^ for self-esteem was statistically significantly associated, *F*(5,417) = 34.30, *p* < 0.001, with global psychological distress over and above the effects of age, parental care, and parental overprotection, (29.1% of the variance in global psychological distress).

## Discussion

This study investigated the prevalence of recall of childhood exposure to parental loyalty conflict behaviors, the kind of parental bonding perceived by adults whose parents had separated or divorced, and the link between parental functioning, individual self-esteem and psychological distress. The current study extends our empirical knowledge of the childhood exposure to dysfunctional parenting in families postdivorce. A number of important findings were shown in the data.

First, 46% of the sample endorsed the item “one parent tried to me against the other parent,” a similar proportion than in Verrocchio and Baker (2013) for the subsample of participants whose parents divorced or separated, indicating the high prevalence of this problem for children of divorce. Results revealed that all behaviors measured by BSQ were endorsed by at least some participants. Rates of endorsement for the specific types of parental behaviors assessed ranged from about 10% (referring to a stepparent as “Mom” or “Dad”) to about 81% (made negative and untrue statements about the other parent to the child). Behaviors endorsed at a particularly high rate included making negative comments about the other parent, indicating discomfort when the child speaks or asks about other parent, confiding in child, and encouraging the child’s reliance. These data are consistent with what is known about the prevalence and the quality of PA within divorcing families (Baker and Chambers, 2011).

Second, a significant finding is that about 65–70% of the participants has perceived by both parents a dysfunctional parenting characterized by low care and high overprotection (affectionless control) or by high care and high overprotection (affectionate constraint) or low care and low overprotection (neglectful parenting). This finding is consistent with specialized literature reporting how separated or divorced parents are less effective in monitoring their children and more emotionally detached from their role as parents (Heatherington and Stanley-Hagan, 1999; Krishnakumar and Buehler, 2000; Wallerstein et al., 2000; Krishnakumar et al., 2003).

Third, the recall of exposure to parental loyalty conflict behaviors varied by parental bonding perceived. Interestingly, parents perceived as less caring and more controlling are also described as more engaged in PA behaviors. This was true for ratings of mothers as well as fathers. This finding provides the empirical confirmation of the theoretical definition of the PA behaviors as a lack of empathy and inability to tolerate the child separate needs and perceptions (Baker, 2007; Baker and Ben-Ami, 2011). Encouraging disobedience in the children and blaming behavior on the other parent, or disparaging the children’s image of the other parent, or sharing excessive information with the child and promoting parentification are all behaviors that involve low care and high psychological control. Likewise, these data contribute to the growing body of knowledge about how rapidly identify parents at risk to engage in alienating behaviors.

Fourth, the results revealed that self-esteem was associated positively with parental care and negatively with parental overprotection and PA. That is, Italian adults’ perception of comfortable bonding with caregivers defined by affection, emotional warmth, empathy, closeness, and promotion of independence and autonomy is associated with a positive self-concept. These results confirm findings from previous studies (Herz and Gullone, 1999; Baker and Verrocchio, 2013) and extend the evidence into Italian adults whose parents are separated or divorced.

The final significant finding was that high exposure to parental loyalty conflict behaviors and low self-esteem were associated with psychological distress even after controlling for quality of the parent–child relationship. Therefore, the current data demonstrate that exposure to parental loyalty conflict behaviors and low self-esteem can also increase an adult’s risk of psychological distress. These data are consistent with literature regarding negative consequences of exposure to PA in adulthood (Baker and Ben-Ami, 2011; Ben-Ami and Baker, 2012; Bernet et al., 2015). The results also support the role of self-esteem as an individual factor to cope with psychological distress (Verrocchio and Baker, 2013) after experiencing negative life events as parental separation/divorce. One possible reason is that low self-esteem could bring a person to perceive only negative aspects of situations or to have a poor perception of one’s capacity to cope with stressing environments.

### Limitations

There are a number of limitations in this study that should be taken into account. The first is the cross-sectional nature of the design. Although this is a common design in studies of PA, we emphasize the need for prospective studies for establishing causal pathways. The second limitation is the retrospective nature of data collected through self-report measures. Data did not necessarily reflect the full complement of parental loyalty conflict behaviors to which the person had been exposed. Nevertheless, Arrindell et al. (1989) reported some overlap between retrospective and independent measures of parental behaviors noting that the perception of the parental relationship is more important than the actual parental relationship. The third is the limited generalizability due to the convenience nature of the sample. The findings should be replicated in other settings and in other samples of various ages, backgrounds, and life experiences.

Despite these limitations, the current study also had several strengths. All of the measures have been used in prior research and most are standardized measures with established reliability and validity. Also, there are methodological advantages of a large community sample used in this study. Furthermore, this was the first study conducted on a general sample of adults whose parents are separated/divorced in Italy highlighting the importance of subsequent research on the causal linkages between dysfunctional parenting and individual resilience and psychological distress. Future research with varied ages and diverse populations as well as other individual variables will complete these findings on how familiar and individual factors affect the adjustment in adulthood following parental divorce.

### Implications

In this study there was strong support for a link between crucial variables to the psychological climate of the divorced families (e.g., parental loyalty conflict, low caring in terms of emotional coldness, indifference, neglect, and high control in terms of overprotection, intrusion, excessive contact, infantilization, and prevention of independent behavior), self-esteem and well-being in adulthood. The results of the present research bring to light several important implications for policymakers and practitioners working with divorced families and suggest the need for interventions based on a greater knowledge of the dysfunctional family processes.

Clinicians and forensic psychologists should be aware that improve parenting practices and promote self-esteem in children or adolescents after marital dissolution may prevent the development of negative outcomes. As can be seen in literature there are many variables (biological, psychological, familiar, interpersonal, etc.) to be considered in the prevention and treatment of psychological distress, but our results about the offspring of divorced parents corroborate the evidence that exposure to PA and individual self-esteem significantly affect their well-being in adulthood.

Prevention programs could be directed to reinforcing health and social services to support divorcing and high conflict families. Health professionals and social workers should receive training specifically about the PA strategies that parents can engage in, dysfunctional parenting practices, and specific negative outcomes for children.

Other prevention programs directed at high risk parents (those divorcing or involved in high conflict) could involve mandatory training for high conflict parents. Psycho-educational programs should focus on interventions for enhancing parenting skills and appropriate child rearing and co-parenting practices. Psychological interventions for children of divorce exposed to dysfunctional parenting could also be carried out to increase coping strategies and resilience in order to protect them from the long-term damage to their sense of self.

Given the potential detrimental impact of parental loyalty conflict behaviors on the well-being of children, it is critical to develop interventions to address this phenomenon. Despite a recent awareness of this problem, very few health and social services use adequate strategies for the management of their clients. Therefore, policy changes in health and social care to promote training on this issue could aid in disseminating of professional training in this area. Practitioners should receive training specifically about how: (a) to create a buffer zone to facilitate crossing the co-parental boundary, (b) to help the child separate from the enmeshment with the alienating parent, (c) to block intrusions from the alienating parent, (d) to strengthen the bond with the targeted parent, and (e) to weaken the coalition around the alienating parent–child dyad (Ellis and Boyan, 2010).

Finally, this information about family dysfunctions in marital dissolution should be widespread to the family court judges in order to decide an arrangement that will promote the best interest of the child.

## Conflict of Interest Statement

The authors declare that the research was conducted in the absence of any commercial or financial relationships that could be construed as a potential conflict of interest.
